# Efficacy of a tip of the big toe remodeling in the distal nail embedding with bone overgrowth of the distal phalanx

**DOI:** 10.1016/j.amsu.2020.08.025

**Published:** 2020-08-30

**Authors:** Mikołaj Dąbrowski, Anna Litowińska, Jolanta Cieślak

**Affiliations:** a Department of Spine Orthopedics and Biomechanics, Poznan University of Medical Sciences, Poznan, Poland; b Anmedica-Healthy Foot Center, Poznan, Poland

**Keywords:** Distal nail embedding, Surgical technique, Osteophyte of distal phalanx, Deformity of hyponychium

## Abstract

**Introduction:**

Distal nail embedding due to hyponychium hypertrophy can be caused by traumatic or surgical avulsion of the nail. As a consequence of these changes, the nail plate is blocked through the deformed tip of the toe. Changes that occur at the tip of the big toe are due to bone growth on the dorsal surface of the distal end of the distal phalanx. This study aimed to present a surgical technique for the treatment of hypertrophy of the tip of the toe and evaluate its effectiveness.

**Material and methods:**

The surgical technique involved remodeling of the tip of the big toe, with removal of the hypertrophied bone of the distal phalanx. The procedure was assessed by using a questionnaire.

**Results:**

We included the 108 distal embedded nails. A total of 85% of respondents were satisfied with the procedure. Nearly 80% of patients rated the cosmetic effect as good or very good.

**Conclusions:**

The technique was an effective treatment and increased the quality of life of those with disorders of nail growth associated with hypertrophy of the tip and hyponychium, with bone overgrowth.

## Highlights

• Distal nail embedding (type of ingrown toenail) is correlated with overgrowth bone.
• The causing distal nail embedding with bone outgrowth is mechanical injury.
• This surgical technique is efficacy (restore normal nail growth) and safety.

## 1 Introduction

The toenail has both protective and sensory functions. The nail plate acts as a support, which has the effect of pressing on the phalanx, increasing the ability to distinguish objects by the skin of the tip. The distal phalanx has an important proprioceptive function, especially when the foot is rolled, and the toe is detached from the ground. Each distal phalanx pathology may have a number of effects that disturb the biomechanics of gait. The absence of a toenail can lead to deformation of the tip of the toe, with simultaneous loss of proprioception [1].

Distal nail embedding is treated as a type of ingrown toenail. It is defined as hypertrophy of the hyponychium. The cause of the change in this area may be congenital or acquired [2], occurring in people of all ages, and causing discomfort or pain of varying intensity. In the case of acquired lesions, the growth of the nail plate can be blocked by the deformed hyponychium [3].

The main cause of distal nail embedding is complete nail detachment for various reasons, either traumatic or surgical, and multiple nail injuries. Other possible predisposing factors are variations in anatomical structure and incorrect positioning of nails.

**Fig. 1:**
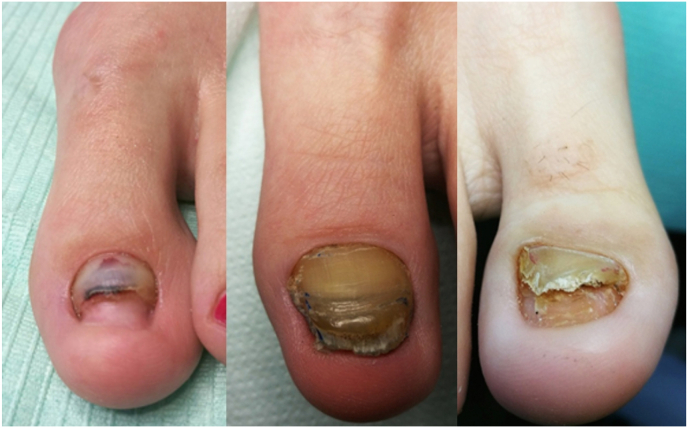
Changes in the toenail due to distal nail embedding with bone spur: lack of growth of the nail plate, chromonychia (discoloration), onychatrophia (deformity, crushing and atrophy), onychomadesis (shedding of the plate from the matrix side), onycholysis, and deformation of the tip of the toe.

Patients undergo the following changes due to the absence of nail plate growth (Fig. 1): chromonychia (change of color), onychatrophia (distortion, crushing, and atrophy), onychomadesis (shedding of the plate from the matrix side), onycholysis, and deformation of the tip of the toe.

The above symptoms are most common for long-term antifungal treatment by dermatologists, and for surgeons to remove the nail plate. In the case of podiatrists, they usually attempt to clean and regenerate the nail bed and possibly taping of the tip of big toe with varying results.

We present a surgical technique and assess its effectiveness in the treatment of hypertrophy of the tip of the toe, caused by earlier injury, microdamage, or removal of the nail plate. We were interested in whether a satisfactory result of this technique. The aim of study is to evaluate the results of removal of the hypertrophied bone of the distal phalanx.

## 2 Materials and methods

### 2.1 Study design

The study was a retrospective study of patients who underwent the procedure with a plastic surgery of the tip of the big toe, with removal of the hypertrophied bone of the distal phalanx. The study was reported in line with the PROCESS criteria [4]. The study was registered under number ChiCTR2000034005 [5].

### 2.2 Participants

Participants in the study were included if they were adolescents or adults who decided to undergo our procedure with removal of the hypertrophied bone of the distal phalanx. Individuals were excluded if they could not understand Polish or had significant medical comorbidities.

Based on clinical volume, we anticipated being able to recruit approximately 80 participants over a two-year period. All participants underwent consultation with one podiatric nurse. Written consent was obtained from the patient; for juvenile participants, written consent was obtained from the parent or legal guardian as well.

**Fig. 2:**
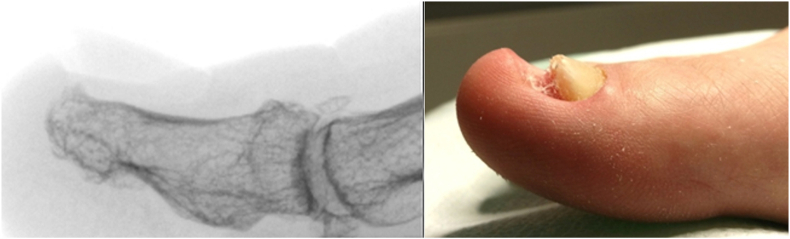
Lateral X-ray image of a bone spur of a distal phalanx with a nail growth problem.

**Fig. 3:**
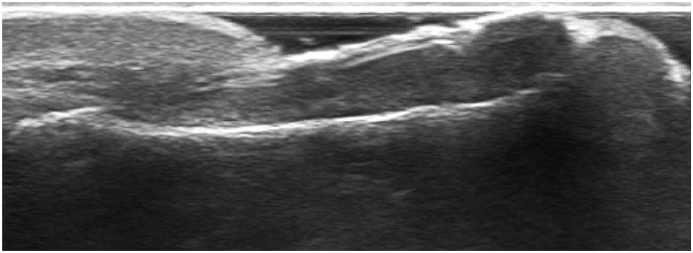
Ultrasound imaging of a bone spur of a distal phalanx with a nail growth problem.

Three patients had hypertension and one a Crohn disease. Other patients did not have comorbidities. Before surgery, patients underwent x-ray diagnostic imaging anteroposterior (AP) and lateral foot views (Fig. 2). In the case of clinical doubt, a pre-operative ultrasound examination of the big toe was performed (Fig. 3). Before surgical intervention, detailed medical histories were obtained from all patients in this case series, and physical examinations were performed. No exclusion criteria were set.

### 2.3 Intervention

All participants underwent the procedure with removal of the hypertrophied bone of the distal phalanx as an outpatient day procedure approximately one to two months after their initial. All surgeries was performed at the outpatient surgical clinic as private practice at Anmedica-Healthy Foot Center (Grunwaldzka Street 121, Poznan, Poland). The procedure was carried out by an orthopedist or surgeon assisted by a nurse, podiatrist. The first author has 8 years of experience in spine surgery (Wiktor Dega Hospital in Poznan, Poland) and in orthopedic surgery and 5 years of experience in nail surgery. The surgeon has many years of experience in general surgery and over 10 years in nail surgery.

### 2.4 Surgical technique

#### 2.4.1 Preparation for the procedure

In order to qualify for surgery, each patient received a health questionnaire, which was sent to the Center, along with photographs. The patients received periprocedural recommendations: an inventory of dressing materials, a method for preparing the toe for surgery, and postoperative procedures, including instructions for dressing changes. They also received recommendations that for three consecutive days before the procedure, the toe should be soaked for 10 min in a prepared solution of potassium permanganate, then dried and lightly bandaged. On the day of the surgery, the patient reported with a toe secured by a bandage.

#### 2.4.2 Anesthesia

First, the involved toe was prepared in a sterile fashion, and ring nerve block anesthesia was applied using 2% lidocaine without epinephrine. After the anesthesia procedure, an elastic tourniquet was placed at the base of the toe to maintain a clear and bloodless surgical field.

#### 2.4.3 Surgical procedure

The operational approach was a modification of the Howard-Dubois procedure [[6], [7], [8], [9]].

**Fig. 4:**
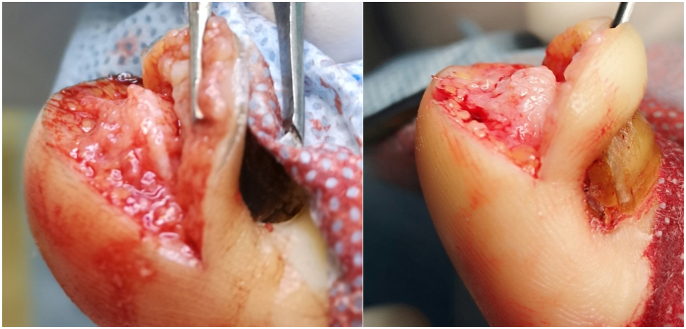
An intraoperative image of a bone overgrowth from the distal phalanx.

**Fig. 5:**
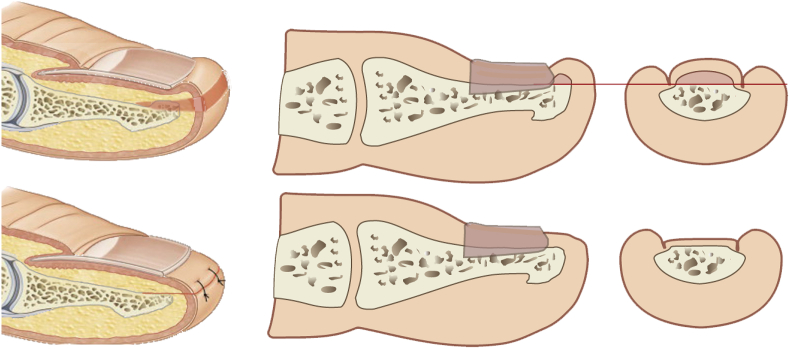
The surgery technique.

A skin incision was made parallel to the tip of the big toe, about 2–5 mm from the highest point of the distal phalanx. The incision began and ended at a height of about 5–8 mm from the proximal part of the nail plate. A second incision was made about 3–5 mm from the first. The skin and subcutaneous tissue were cut in the form of a wedge and removed. Then, the distal end of the distal phalanx at the dorsal surface was dissected. The bone spur was exposed (Fig. 4, Fig. 5) and removed with a bone rongeur, and then the dorsal surface of the distal phalanx was rubbed using a surgical high-speed drill with a flat-ended taper bur. The next stage was optimum remodeling of the tip of the toe. We used single stitches every 3–4 mm. A sterile dressing, which was removable by the patient after 48 h, was placed. The patient changed subsequent dressings every 24 h. The average duration of surgery was approx. 25–40 min, including the waiting time for anesthesia.

#### 2.4.4 After surgery

Patients were discharged home about 30 min after surgery. They received an information card and recommendations for handling the wound. If the surgery involved two toes, the patient was given 50 mg ketoprofen orally after the first toe operation. In the case of blood congestion of the dressing, a further layer of dressing was applied. For two days after the operation, patients were instructed not to soak the surgical area in water. Patients could wash, provided the involved foot remained outside the bath or shower. They were also informed that they should not overload the forefoot for the first three days; however, they could and should walk. Until the day the sutures were removed (about 12–14 days), wearing of full or tight shoes was not recommended. After about 48 h, the first dressing change occurred, with subsequent changes every 24 h. If there was no excessive bleeding or exudate, the dressing was gradually decreased. For 3–5 days after removal of the stitches, the patient continued to use dressings. On subsequent days, the use of an anti-scarring preparation was mandatory. To facilitate complete healing, using a swimming pool, sauna, or solarium; participating in sports; and wearing tight shoes were not allowed. At about 48 h, when the patient changed the bandage for the first time, control photographs of the surgical area were taken and sent to the Clinic by email.

From the moment of full healing, the patient started therapy to control the growth of the nail plate, regenerate the nail bed, prevent scarring, and shape the tip of the toe. For this purpose, a preparation for scars, which improved the condition of the nail bed and accelerated the growth of the nail, was used. In some cases, orthonyxia (nail shape control) and tapping of the tip was performed.

### 2.5 Outcomes

The primary outcome for the study was satisfaction of surgery and recurrence. To assess the surgical procedure and obtain clinical information, we used a online questionnaire.

The standardized questionnaire mailed to all patients involved patient self-assessment, with responses recorded on a 7-point Likert scale. The questions were scored with a value of 1 for “strongly disagree” and a value of 7 for “strongly agree.” In cases where the results were taken as aggregates, values 1–3 were assigned to “agree”, values 5–7 were designated “disagree”, and a value of 4 was considered “neutral”. The questions concerned the patient's satisfaction with the treatment:

How do you assess your satisfaction with the surgery?

How do you assess the aesthetic result after surgery?

How do you assess the appearance of the nail plate after the procedure?

How do you assess the appearance of the postoperative scar?

Is there any abnormal innervation of the tip of the toe?

In addition, four questions were asked with possible answers being either “yes” or “no”:

Is there a problem with the growth of the nail?

Has the problem you had surgically corrected reappeared at the same location? Was further medical or surgical treatment required in the area where the surgery was performed?

Complications occurred in three toe operations. Complications were assessed by asking to participants to record any clinical sequelae related to surgery that prompted a phone call to the surgeon's office, additional clinic appointment or visit to the emergency department. The question concerned the patient's complication with the treatment: Have you experienced complications?

### 2.6 Statistical analysis

Statistica 13.0 software (StatCorp., College Station, TX, USA) and Microsoft Excel 2016 were used in the analysis. Descriptive values of variables were expressed as means ± standard deviation or medians (minimum-maximum). Statistical significance was set at p < 0.05. Dichotomous data were presented as counts and frequencies and continuous data as means and standard deviation (SD). To compare the impact of various factors, a *t*-test was performed.

## 3 Results

### 3.1 Baseline characteristics

We retrospectively reviewed charts 108 distal embedded nails with bone spurs were operated on in 76 patients between 2016 and 2018. The average follow-up time was 1.5 years.

Women predominated (86.8%), and the average age of the patients at surgery was 31.6 years. Patients had had the deformation for an average of 7.1 years, with two patients having a distal embedded nail for up to 20 years. The mean age of patients who answered questions about the effects of treatment was 35.9 years, and 84.2% were female (Table 1). We collected data on previous treatment concerning 80 toes in 57 (75%) patients.

**Table 1:** Characteristics of the study group.

Parameter		
Age [Years]	Mean ± SD	31.6 ± 10
	Median (min-max)	29 (16–69)
Sex	Female	66 (86.8%)
	Male	10 (13.2%)
Duration of deformation of the big toe [years]	Mean ± SD	7.1 ± 4.6
	Median (min-max)	8 (1–20)
Observation time [years]	Mean ± SD	1.6 ± 0.7
	Median (min-max)	1.4 (0.5–2.6)
Toes operated on	Unilaterally	
	Right	30 (39.5%)
	Left	14 (18.4%)
	Both sides	32 (42.1%)
Cause of bone spur – mechanical trauma	Yes	54 (71.1%)
	No	19 (25.0%)
	I don't remember	3 (3.9%)
Previous treatment	Surgery – removal of the nail	48 (63.2%)
	Dermatologist – antifungal treatment	48 (63.2%)
	Nail claws	30 (39.5%)
	Arcada's cube	3 (3.9%)
	Nail plate reconstruction	12 (15.8%)
	Not treated	5 (6.6%)

SD, standard deviation.

### 3.2 Recurrence

Our questionnaire defined recurrence as “further medical or surgical treatment required in the area where the surgery was performed.” The question was direct and required a “yes” or “no” answer. The responses to this question were directly correlated with the finding that no patients experienced a direct recurrence(Table 2) (Fig. 8)

**Table 2:** Numbers and percentage shares of responses to questions regarding the operative treatment of bone spurs in patients (n = 80 toes).

Is there a problem with the growth of the nail?	Yes	22 (27%)
	No	58 (73%)
Has the problem you had surgically corrected reappeared at the same location?	Yes	5 (6%)
	No	75 (94%)
Was further medical or surgical treatment required in the area where the surgery was performed?	Yes	0
	No	80 (100%)
Have you experienced complications?	Yes	9 (11%)
	No	71 (89%)

We further sought to ensure appropriate responses by asking patients if “the problem you had surgically corrected reappeared at the same location.” In our cohort, 94% reported that the problem had not recurred.

For the question “Is there still a problem with the growth of the nail?“, 73% of patients responded that they no longer had a problem.

### 3.3 Patient satisfaction

Overall, 85% of respondents to the questionnaire strongly agreed that they were satisfied with the procedure, and none of the patients were highly dissatisfied with the surgery. One patient with surgery on two toenails reported a value of 2 on the 7-point scale. A score of 7 was recorded by 48% of patients, while 22% responded with 6 on the 7-point scale.

Only where two toenails were operated on was the aesthetic effect unsatisfactory. Such patients assessed the cosmetic effect at 7 points (48%), 6 points (26%), and 5 points (7%).

**Fig. 6:**
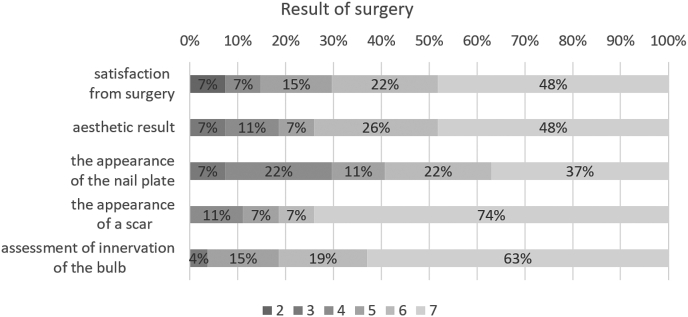
Percentage of answers to questions about patients' satisfaction with surgical treatment Legend: 1 = “strongly disagree” and 7 = “strongly agree”.

**Fig. 7:**
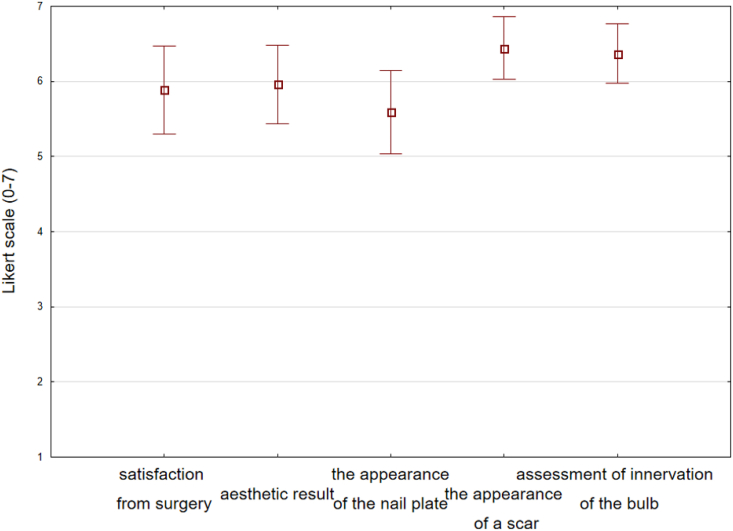
Postoperative responses of patients who underwent surgical bone spur removal. Graph of the mean values and standard deviations of the answers to questions about satisfaction with the surgery.

**Fig. 8:**
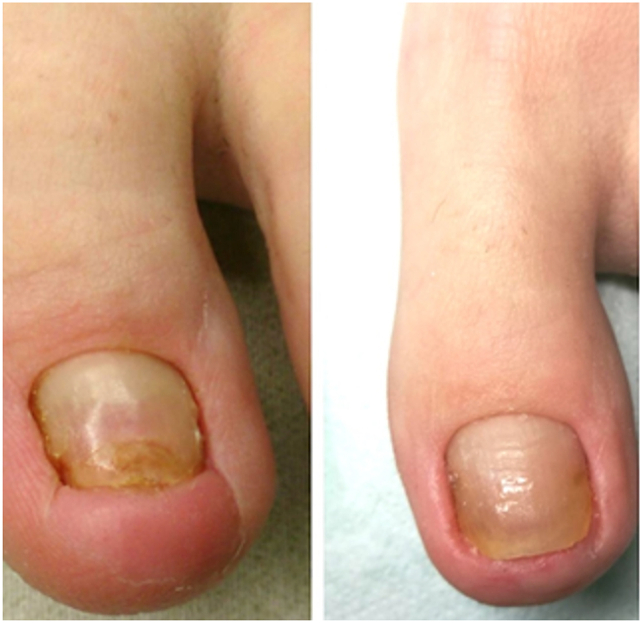
Clinical outcomes of treatment before and after surgery.

Most patients (93%) had no problems with the appearance of the scar Fig. 8. Patients had the most concerns about the appearance of the nail plate, though 37% were very satisfied (7 points), 22% satisfied (6 points) and 11% recorded 5 points, nearly 30% of patients assessed the nail plate at 3 (7%) and 4 points (22%)Fig. 6, Fig. 7. These last responses were consistent with the 33% who reported the further occurrence of nail growth disorders (Table 2.).

### 3.4 Complications

**Fig. 9:**
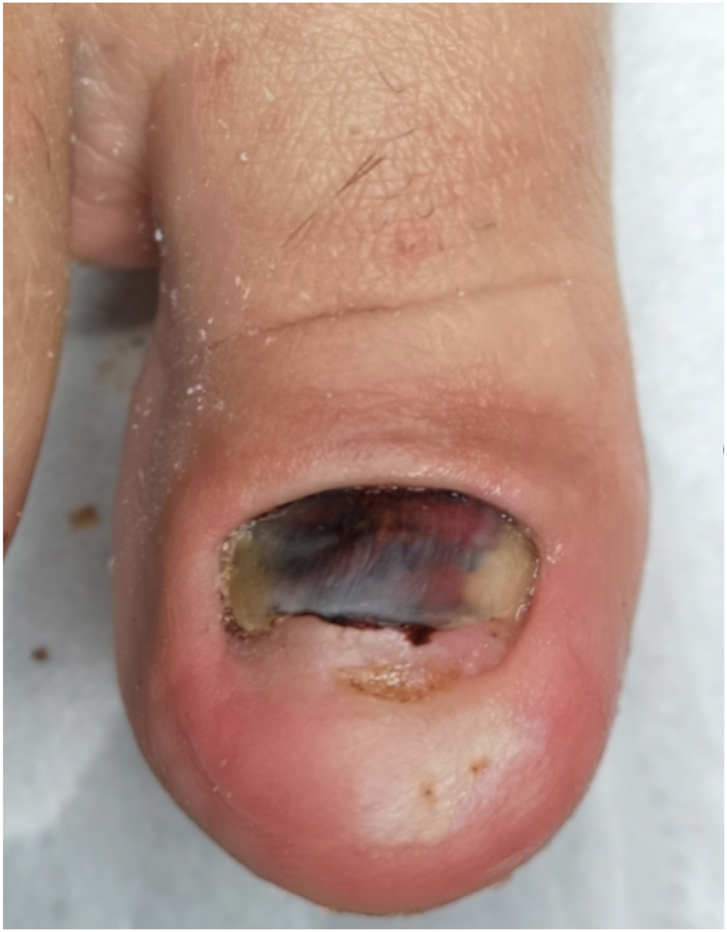
Subnail hematoma after embedded distal nail surgery.

**Fig. 10:**
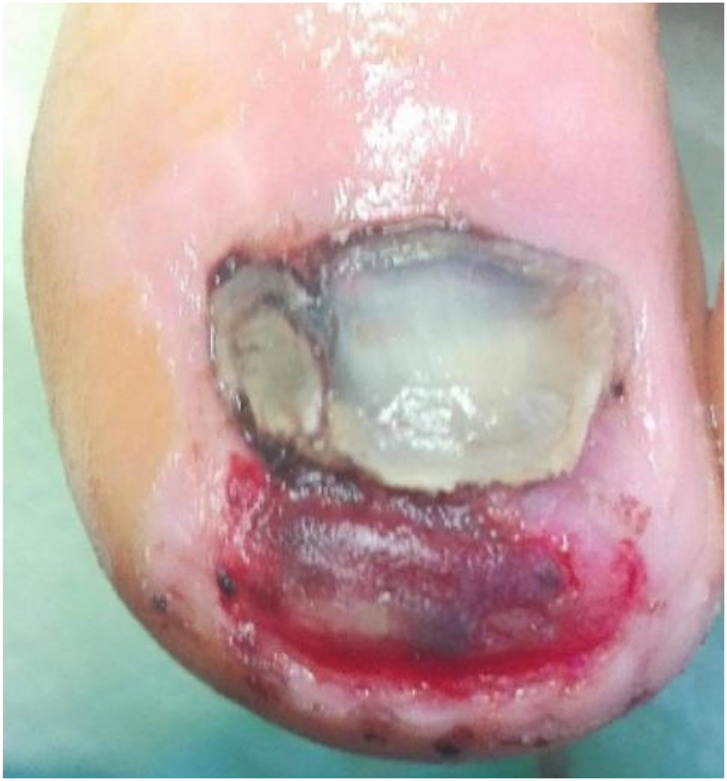
Hematoma at the treatment site and prolonged healing.

Complications occurred in three toe operations. The most common post-operative problems were postoperative hematomas and prolonged healing (Fig. 9, Fig. 10). In five cases there was necrosis of the skin, which subsequently healed.

An early infection of the surgical site occurred in one patient with inflammatory bowel disease, which influenced nutrition and healing and likely disrupted antibiotic absorption. At an early stage, the surgical wound was cleaned, and further healing proceeded without complications.

One patient developed healing in the form of a keloid, which partially affected nail growth.

Abnormal innervation of the tip of the toe occurred in four patients who responded to the questionnaire with an assessment of 3 out of 7 points.

## 4 Discussion

### 4.1 The process of creating a bone outgrowth

**Fig. 11:**
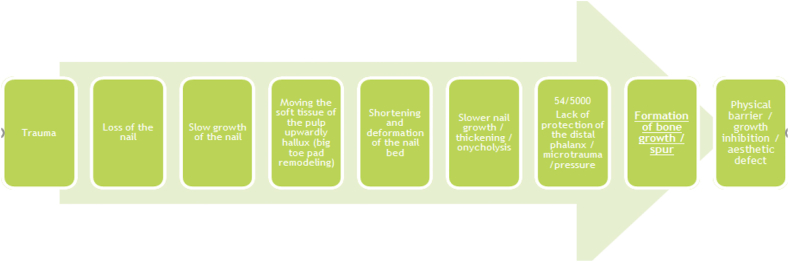
Probable sequence of changes leading to the formation of a bone outgrowth.

The probable sequence of changes leading to the formation of a bone outgrowth is presented in Fig. 11. Distal nail embedding of the toenail is a typical consequence of either too short a nail or the lack of a nail, particularly after nail avulsion. Due to the lack of counterpressure, the tip of toe is dislocated dorsally when the foot rolls during gait. Creates a distal obstruction that blocks the growth of the nail plate. The loss of back pressure caused by the loss of the nail plate leads to a widening of the dorsal part of the tip of the toe. There is a remodeling of the distal part of the toe and subsequent hyperkeratotic reaction. There is a shift in the soft tissues of the big toe pad and its remodeling. As a result, the nail bed is shortened and deformed. At a later stage, the nail growth decreases, the thickness of the nail increases, and possible onycholysis occurs [1]. Lack of protection of the distal phalanx increases its susceptibility to micro-injuries and pressure.

X-ray and ultrasound confirm that soft tissues of the distal toe, may remodeling under the influence of micro-injuries. In addition, some kind of bone protection may be stimulated in the form of a bone outgrowth.

According to normal anatomy, the superior surface of the distal phalanx of the big toe is flat longitudinally and convex transversely and extends to the anterior tip of the phalanx. The superior surface is very smooth and is covered by nail bed mesenchyme [10].

Current views on the mechanism of formation of osteophytes define them as bone growths that develop at the edges of joints with osteoarthritis [11]. Histologically, osteophytes resemble a epiphyseal growth plate, with both regions showing chondrocyte hypertrophy, new cartilage formation, and vascularization leading to bone ossification. It is believed that neovascularization is stimulated by vascular endothelial growth factor (VEGF), which is expressed by hypertrophic chondrocytes. Antiangiogenic therapies may reduce marginal bone growth in animal models of arthritis, although the significance of this new bone in terms of pain remains undetermined [12].

Naming of this bone spur with osteophytes is controversial, although Heneke used the term “distal dorsal osteophyte” in the description of changes in the distal phalanx of the toe associated with pincer nail [13]. In our opinion, it should be called distal dorsal digital bone outgrowth/spur formation, because the forming bone is not directly in a joint. As such, the concept of exostosis should be avoided, because it may mistakenly suggest exostosis subungualis, which is defined as a slow growing, benign outgrowth of the normal bone under the nail that affects the nail unit. The most common location of bone outgrowths is in the distal phalanx of the big toe [14].

Piracicini et al. described 15 cases of children who had a nail removed due to retronychia [15]. Five of the 13 patients had persistent nail dystrophy, in the form of a bold yellowish toenail, which grew very slowly. The symptoms described in their report suggested distal nail embedding, with bone spur of the distal phalanx of the toe. In such patients, often with many years of nail plate changes, dermatologists often recognize onychomycosis, although this is not indicated by mycological studies. They often start long-term antifungal therapy, which in most cases is ineffective. This procedure was found in 60% of patients in the current study. Ineffective dermatological treatment or surgical removal of the nail was found to further aggravate the existing deformity. In addition, surgical operations where the nail was removed did not bring about the intended effect, most often leading to an even lower increment of nail growth.

From the analysis of the cases in the current study, it was clear that the main factor causing distal nail embedding with bone outgrowth was mechanical injury (sport, tight shoes, mountain climbing) or complete removal of the nail in the surgical treatment of ingrown nails.

When dermatological treatment and surgical removal of the nail are ineffective, this surgical technique should be uses.

### 4.2 Surgery technique

Until now, the name “distal nail embedding” has been used to describe a type of ingrown nail. There were no changes in the distal part of the toe with osteophyte formation. Chang et al. reported 24 cases with a typical long-term history of antifungal treatment, in which surgery by the Dubois method was suggested [16,17]. However, the Howard-Dubois method removes only soft tissues [7].

Careful attention should be paid to preparation of the skin on the dorsal part of the phalanx for osteonecrosis of the bone. This is an extremely important stage in the entire surgical procedure and affects the healing process. In some cases, it is extremely easy to break the continuity of the skin, resulting in hematoma, necrosis of the upper flap, and longer healing times.

Our experience showed that it was very important to protect the lower limb and forefoot in the first days after surgery. Postoperative hemorrhage naturally arises at the surgical site. However, too rapid a return to full activity by the patient may contribute to maintenance or escalation of vascular stasis, longer healing times, and worse treatment outcomes.

In this surgical technique, it was important not to remove too much subcutaneous tissue. This observation was made in other centers in Poland, where a significant reduction of tissues, especially fatty and subcutaneous tissues, was made. This led to severe cosmetic effects and pain, and the skin became partially necrotic with dystrophy, bruising, and thus long-lasting convalescence.

One postoperative complication that can occur following excision of soft tissue is a loss of cutaneous innervation. In our population, 63% of patients reported that they did not have any loss of sensation around the operative site. However, 4% stated that they had significant loss of sensation around the surgical area.

### 4.3 Satisfaction with the procedure

Evaluation of the aesthetic results and the surgery seemed to recognize the procedure present in this current study as effective.

The problem of poorer plaque growth in a large group of patients after surgery (about 30%) might have resulted from several complications, including prolonged nail deformation before surgery and, as we observed, a lack of compliance by patients after the procedure. From our experience, the postoperative process was as important as the operation. A podologist was indispensable not only to help diagnosis the problem but also to coordinate the process of healing and stimulation of nail growth post-surgery. A qualified podologist can recognize the reason for the lack of nail plate growth. The basis for diagnosis is a history of treatment, previous surgical procedures, medications and preparations used, palpation examination, X-ray imaging, and consultation with the clinic performing the surgical treatments.

Postoperative therapy involves controlling the growth of the nail plate and the condition of the surgical site. Every 3–4 weeks, the podologist cleans the toenail and surgical site, checking whether the patients are following the care instructions.

The patient uses preparations to accelerate regeneration of the nail bed after removal of sutures and full healing of the tip of toe, performing massages to increase tissue congestion. During follow-up visits, the podologist checks the condition of the nail bed, its hydration, and the degree of increase in the distal direction.

Control of nail growth includes its cleansing and, in some cases, the use various types of orthonyxia, in order to give the correct shape and direction of nail plate growth. The most commonly used and recommended orthonyxia is a titanium clamp.

Cooperation with a physiotherapist helps to provide the patient with orthopedic insoles, correcting posture and positioning of the foot.

Patient education is a very important element of therapy, aimed at maintaining the effect of surgery. The patient receives information on proper footwear, prevention of injuries and procedures in the event of a nail injury, physiotherapy tips, care instructions, and information that should alert the patient to a problem with the nail.

Further research is aimed at increasing the group of patients with repeated assessment of treatment satisfaction and at the same time extending the observation time. Second topic of research is to develop methods of dealing directly after surgery reducing the risk of hematoma.

### 4.4 Limitations

There are several limitations to this study. The sample size was small and, due to low statistical power, the results may not show all possible differences in statistical significance. In addition, the used treatment satisfaction questionnaires present a subjective assessment of patients. There is no objective clinical assessment of the condition of the nail after surgery.

## 5 Conclusions

This surgical technique described in this study was an effective treatment in cases of nail growth disorders associated with hypertrophy of the tip and hyponychium of the toe with bone spur or bone overgrowth. Diagnostics should be extended by X-ray or ultrasound to confirm the occurrence of a bone spur or bone overgrowth at the distal end of the distal toe. Information requested at the clinical interview should include that on microtrauma or previous removal of the nail, e.g., due to an ingrown nail. In addition, ineffective use of antifungal treatment might also aid in the diagnosis. We believe that this surgical technique, which has been successfully used in Poland for several years, will help the patients with long-term problems and, at the same time, improve their quality of life.

## Ethical approval

Poznan University of Medical Science no 33/20.

## Sources of funding

None.

## Author contribution

Conceptualization – MD, AL, JC. Data curation – MD. Formal analysis – MD. Funding acquisition – AL. Investigation – MD. Methodology – MD, AL, JC. Project administration – MD, AL. Supervision – AL. Validation – AL. Roles/Writing of original draft – MD. Writing review & editing – MD, AL.

## Trial registry number

1. Name of the registry:
  Chinese Clinical Trial Registry.
2. Unique Identifying number or registration ID:
  ChiCTR2000034005
3. Hyperlink to your specific registration (must be publicly accessible and will be checked):
  http://www.chictr.org.cn/showprojen.aspx?proj=55533

## Guarantor

Mikolaj Dabrowski

## Consent

Written informed consent was obtained from the patient for publication of this case report and accompanying images. A copy of the written consent is available for review by the Editor-in-Chief of this journal on request.

## Ethics

The work was approved by the appropriate ethical committees related to the institution(s) in which it was performed. The patients provided informed consent to the work.

## Provenance and peer review

Not commissioned, externally peer reviewed.

## Declaration of competing interest

None.
